# Preoperative Prediction of Microvascular Invasion in Hepatocellular Carcinoma: Initial Application of a Radiomic Algorithm Based on Grayscale Ultrasound Images

**DOI:** 10.3389/fonc.2020.00353

**Published:** 2020-03-19

**Authors:** Yi Dong, Liu Zhou, Wei Xia, Xing-Yu Zhao, Qi Zhang, Jun-Ming Jian, Xin Gao, Wen-Ping Wang

**Affiliations:** ^1^Department of Ultrasound, Zhongshan Hospital, Fudan University, Shanghai, China; ^2^Suzhou Institute of Biomedical Engineering and Technology (CAS), Suzhou, China

**Keywords:** hepatocellular carcinoma (HCC), ultrasound, machine learning, algorithm, microvascular invasion (MVI)

## Abstract

**Objectives:** To establish a radiomic algorithm based on grayscale ultrasound images and to make preoperative predictions of microvascular invasion (MVI) in hepatocellular carcinoma (HCC) patients.

**Methods:** In this retrospective study, 322 cases of histopathologically confirmed HCC lesions were included. The classifications based on preoperative grayscale ultrasound images were performed in two stages: (1) classifier #1, MVI-negative and MVI-positive cases; (2) classifier #2, MVI-positive cases were further classified as M1 or M2 cases. The gross-tumoral region (GTR) and peri-tumoral region (PTR) signatures were combined to generate gross- and peri-tumoral region (GPTR) radiomic signatures. The optimal radiomic signatures were further incorporated with vital clinical information. Multivariable logistic regression was used to build radiomic models.

**Results:** Finally, 1,595 radiomic features were extracted from each HCC lesion. At the classifier #1 stage, the radiomic signatures based on features of GTR, PTR, and GPTR showed area under the curve (AUC) values of 0.708 (95% CI, 0.603–0.812), 0.710 (95% CI, 0.609–0.811), and 0.726 (95% CI, 0.625–0.827), respectively. Upon incorporation of vital clinical information, the AUC of the GPTR radiomic algorithm was 0.744 (95% CI, 0.646–0.841). At the classifier #2 stage, the AUC of the GTR radiomic signature was 0.806 (95% CI, 0.667–0.944).

**Conclusions:** Our radiomic algorithm based on grayscale ultrasound images has potential value to facilitate preoperative prediction of MVI in HCC patients. The GTR radiomic signature may be helpful for further discriminating between M1 and M2 levels among MVI-positive patients.

## Key Points

- A radiomic algorithm based on grayscale ultrasound images has potential value to facilitate preoperative prediction of MVI in HCC patients.
- Gross-tumoral region (GTR) and peri-tumoral region (PTR) signatures were combined to generate gross- and peri-tumoral region (GPTR) radiomic signatures.
- The GTR radiomic signature may be helpful for further discriminating between M1 and M2 levels among MVI-positive patients.

## Introduction

Hepatocellular carcinoma (HCC) is one of the most common type of liver malignancies all over the world and exhibits aggressive malignant behavior and a high mortality rate (1, 2). For HCC patients, hepatic surgery is the primary treatment, but 5-years recurrence rates after hepatic surgery could be as high as 50% (1, 2), which varies from 20 to 44% (3). Therefore, it is important to make pre-operative risk stratification of early recurrence for optimizing patient management.

In recent years, microvascular invasion (MVI) has been proved to be an independent predictor of poor outcomes subsequent to surgical hepatic resection (4–6). Currently, MVI status cannot be adequately determined or predicted preoperatively, and the only method to determine MVI status is via postoperative histopathology (4). Therefore, to make non-invasive and accurate identification of MVI preoperatively would be of great benefit for stratifying HCC patients before surgery (4, 7, 8).

Preoperative serum tumor markers and gene signatures have been investigated as possible approaches for the prediction of MVI (5, 9). However, such methods are relatively complicated and the prediction results are indirect, which have not yet been validated or routinely applied in daily clinical practice (10). Extensive studies have been proposed to use various imaging methods to predict MVI in HCC. Current reports of MVI classification have been mainly based on computed tomography (CT) (11–13), magnetic resonance imaging (MRI) (14–16), and contrast-enhanced ultrasound (CEUS) (17, 18). Several imaging features have been proposed as predictors of MVI, such as the status of tumor-internal arteries, hypodense halos on CT scans, arterial peritumoral enhancements, non-smooth tumor margins, and peritumoral hypointensities on gadoxetic-acid-enhanced MRI (16). In combination with the numbers and sizes of tumors, CEUS washout rate may have a role in identifying HCC patients with MVI (17). However, such qualitative radiological characteristics have been based on subjective evaluation by individual radiologists and lack high-dimensional features from different frequency scales. Unfortunately, no current imaging methods could make a direct and accurate diagnosis of MVI based on imaging features (19, 20).

The radiomic method is a brand new imaging technique with the assistance of artificial intelligence software in performing high-throughput extraction of advanced quantitative features (21–23). By extracting high-dimensional features to quantify tumor heterogeneity from radiological images, preoperative MVI assessment in HCC can be hopefully realized (22, 24–27). Previous studies have shown that radiomics may potentially be applied via CT and MRI in classification of HCC grades, early recurrence prediction, and evaluation of biological characteristics in HCC patients (15, 18, 21, 28–30). Ma et al., established radiomic signatures based on contrast-enhanced CT to predict the status of MVI (11). Yang et al., constructed radiomic signatures based on MRI for prediction of MVI (14). However, CT and MRI still have limitations, such as CT having a potential risk of radiation exposure, and MRI being relatively expensive and time consuming.

Grayscale ultrasound is the most commonly used first-line imaging method of HCC lesions before operation, which has unique advantages in terms of being a non-radiation, easy-to-perform, and cost-effective imaging method. Recent studies have shown that radiomic analysis can also be applied to ultrasound images (11, 14). Radiomic scores based on ultrasound images have potential to non-invasively predict the MVI status in HCC patients (18). In a previous study, the imaging features of CEUS for assessment of MVI were evaluated preoperatively. However, none of the qualitative CEUS features were proved to be directly associated with MVI (18).

Preoperative assessments of MVI via various imaging modalities mainly focused on features inside of tumor, while the peri-tumoral areas have been less explored. Pathologically, peri-tumoral areas is the first area of incidence of MVI. It acts as the main blood dissemination path to portal venous thrombosis, as well as metastases in both intrahepatic and extrahepatic areas (31). Therefore, comparing to the tumor area, imaging features involving peri-tumoral areas may reveal a more direct association with MVI (23).

In our present study, we aimed to establish a radiomic algorithm based on grayscale ultrasound in both tumoral and peri-tumoral areas and to make preoperative predictions of MVI in HCC patients.

## Materials and Methods

### Institutional Board Approval

This retrospective study was approved by the institutional review board of our institution. Informed consent was waived before ultrasound examination. All procedures were in accordance with the Declaration of Helsinki.

### Patients

The inclusion criteria were as follows: (1) grayscale ultrasound imaging was performed preoperatively in each patient; (2) no prior surgical or medical treatment was administered for the suspected HCC lesions; (3) hepatic resection was performed within 2 weeks after preoperative ultrasound imaging; and (4) diagnoses of HCC were confirmed by surgical resection and histopathological results.

The exclusion criteria were: (1) patients received locoregional therapy (i.e., radiofrequency ablation or trans-arterial chemoembolization) before ultrasound imaging; (2) Focal cystic liver lesion; (3) unclear or unsatisfied ultrasound images of focal liver lesions.

Following screening based on inclusion and exclusion criteria, 322 patients were enrolled from January 2016 to December 2018. The mean time interval between ultrasound imaging and surgery was 10 ± 1 days. The clinical characteristics of patients—such as patients' age, gender, tumor maximum diameter, serum carcinoembryonic antigen (CEA) values, alpha-fetoprotein (AFP) values, and carbohydrate antigen 19-9 (CA19-9) values—are recorded [Table 1]. Differences in variables were assessed by using the independent Wilcoxon rank-sum test for continuous variables. For categorical variables, the chi-square test was performed. The statistical significance set at 0.05 (two-sided).

**Table 1:** Baseline characteristics of patients.

**Characteristic**	**HCC MVI (-) (*n* = 178)**	**HCC MVI (+)** **(*n* = 144)**	***P-*value**
Age (year)			0.304
Mean ± SD	58 ± 11	57 ± 9	
Range	20–81	29–74	
Male/female	143/35	129/15	0.037
**Etiology of liver disease**			
Hepatitis B	137	120	
Hepatitis C	3	5	
Alcohol	1	0	
NAFLD	12	5	
Absence	25	14	
AFP (ng/l)	28 ± 10	506 ± 8	<.001*
CA 19-9	38 ± 7	425 ± 19	0.784
CEA	4.8 ± 1.3	9.7 ± 5.5	0.635
Tumor size (mm)			<0.001*
Mean ± SD	32.3 ± 23.3	48.4 ± 30.6	
Range	9–144	6–176	

*HCC, hepatocellular carcinoma; MVI, microvascular invasion; AFP, Alpha-fetoprotein; CEA, carcinoembryonic antigen; CA19-9, carbohydrate antigen 19-9; NAFLD, non-alcoholic fatty liver disease*.

### Ultrasound Examination Procedure

Grayscale ultrasound examinations were performed by three experienced radiologists (more than 10 years of experience in liver ultrasound scans) who were aware of the patients' clinical histories. Standardized ultrasound image acquisition procedure were performed 2 weeks before operation. The imaging parameters were adjusted and optimized for each image, including (1) brightness gain set between 80 and 90%; (2) depth set between 10 and 15 cm; (3) dynamic range set between 65 and 80 dB; (4) the HCC lesion was set in the center of field of view during ultrasound scan; and (5) the focal zone was set in the bottom area of image.

Ultrasound examination was performed by using one of the following ultrasound machines: LOGIQ 9 (GE Healthcare, United States; C1-5 convex array probes, 1–5 MHz); LOGIQ E9 (GE Healthcare, United States; C1-5 convex array probes, 1–5 MHz); Acuson Sequoia 512 (Siemens Medical Solutions, United States; 6C1 convex array probes, 3.5 MHz); S2000 HELX OXANA unit (Siemens Medical Solutions, Germany; 6C1 convex array probes, 3.5 MHz); S3000 HELX unit (Siemens Medical Solutions, Germany; 6C1 convex array probes, 3.5 MHz); Philips IU 22 (Philips Bothell, United States; C5-1 convex array probes, 1–5 MHz); EPIQ7 unit (Philips Bothell, United States; C5-1 convex array probes, 1–5 MHz); Aplio XV (Toshiba Medical systems, Japan; PV1-475BX probe, 1–8 MHz); and Aplio i900 series diagnostic ultrasound system (Cannon Medical systems Corporation, Japan; PV1-475BX probe, 1–8 MHz).

For each HCC lesion multiple slices were acquired and recorded, among which the best one was selected for further radiomics analysis. The criteria of ultrasound image selection were as follows: (1) maximum diameter of the lesion; (2) the margin of the lesion was clear and (3) the surrounding liver parenchyma of the lesion was clearly scanned. In order to reduce the influence of image acquisition variants, two radiologists with more than 10 years of liver ultrasound operating experience reviewed all ultrasound images and excluded unqualified slices.

### Histopathologic Examination of MVI

All hepatic specimens were reviewed by a hepatic pathologist with more than 15 years of experience in hepatic pathology. The pathologist was blinded to clinical information or preoperative ultrasound findings. The histopathological diagnosis of MVI was made according to the Practice and Guidelines of the Chinese Society of Pathology. Three subgrades of MVI included the following: M0, no MVI; M1 (the low-risk group), ≤ 5 MVI in adjacent liver tissue and ≤ 1 cm from the tumor; and M2 (the high-risk group), > 5 MVI or MVI in liver tissue and > 1 cm from the tumor (32).

### Workflow of Radiomic Analysis

**Figure 1:**
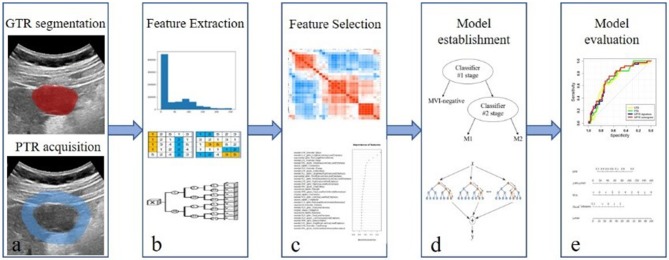
Workflow of radiomic analysis. The workflow of radiomic analysis included the following: **(a)** tumor segmentation; **(b)** feature extraction; **(c)** feature selection; **(d)** radiomic model establishment; and **(e)** model evaluation.

The workflow of radiomic analysis included the following: (1) tumor segmentation; (2) feature extraction; (3) feature selection; (4) radiomic model establishment; and (5) model evaluation (Figure 1).

In our present study, the classification was performed in two stages. MVI-negative and MVI-positive cases were classified during the classifier #1 stage. MVI-positive cases were further classified as either M1 or M2 at the classifier #2 stage. For the classifier #1 stage, 221 cases were examined via six different ultrasound machines and were used as the training cohort, and the remaining 101 cases were examined via three other ultrasound machines and were selected as the validation cohort. For the classifier #2 stage, 107 cases were examined via four different ultrasound machines and were used as the training cohort, and the residual 37 cases were examined via three other ultrasound machines and were selected as the validation cohort.

#### Step 1: Tumor Segmentation

**Figure 2:**
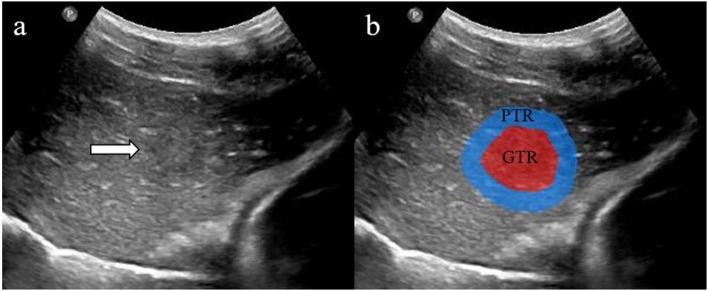
Two regions of interest (ROIs) were defined in grayscale ultrasound images **(a)**. The red area shows gross-tumor region (GTR) signatures, and the blue area shows peri-tumoral region (PTR) signatures **(b)**.

For each HCC lesion, the segmentation of the gross-tumor region (GTR) was accomplished by an experienced ultrasound radiologist (with 15 years of experience) using the Medical Imaging Interaction Toolkit (MITK; version 2013.12.0; http://www.mitk.org/), which was confirmed by another radiologist (with 8 years of experience). The uniform dilated half of the tumor radius served as the peri-tumoral region (PTR) along the border of GTR (Figure 2).

#### Step 2: Feature Extraction

Since nine ultrasound machines were involved in this study, imaging normalization calculated by z-scores was applied to achieve a zero mean and unit variance based on each ultrasound machine. The radiomic features of both GTR and PTR at the classifier #1 stage and classifier #2 stage were extracted using PyRadiomic radiomic toolbox (33). The full intensity range of each region of interest (ROI) was quantized to 32 gray levels, and the normalization scale was set as 255. The radiomic features were divided into three classes: 14 morphological features, 306 first-order statistical features, and 1,275 textural features. The radiomic features were further extracted based on five gray matrices that included the gray-level co-occurrence matrix (GLCM), gray-level size-zone matrix (GLSZM), gray-level run-length matrix (GLRLM), gray-level dependence matrix (GLDM), and neighborhood gray-tone difference matrix (NGTDM). In addition, seven imaging filters were applied to the original imaging datasets in order to extract high-dimensional features from different frequency scales and included the following: wavelet, square, square root, logarithm, exponential, gradient, and local binary pattern (LBP) filters. Finally, 1,595 quantitative radiomic features were extracted from each ROI. A detailed description of radiomic features is provided in Supplement A.

#### Step 3: Feature Selection and Classifier Modeling

In order to eliminate redundant features, Pearson correlation analysis was performed to calculate the pair-wise feature correlation (34). The features with a mean absolute correlation higher than 0.9 were considered to be redundant and were thus eliminated (35). After the elimination of redundant features, we used a feature-ranking algorithm (minimum redundancy maximum relevance, mRMR) (36) to select the most important features based on a heuristic scoring criterion. Ultimately, the top ranked features were selected.

#### Step 4: Radiomic Model Establishment

A random forest (RF) (37) was employed to establish radiomic signatures using the top-ranking radiomic features from both GTR and PTR in our present study. Subsequently, GTR and PTR radiomic signatures in two classifier stages were generated.

In addition, classifiers were trained using 10-fold cross-validation to determine the optimal parameter configuration on the training cohort. The GPTR signatures were developed on features extracted from the combined region of GTR and PTR. Finally, an integrated signature denoted as the gross- and peri-tumoral volume (GPTR) signature was generated by logistic regression using GTR and PTR signatures. The optimal radiomic signature with the highest area under the curve (AUC) was selected.

The radiomic algorithm was built by multivariable logistic regression, which incorporated the optimal radiomic signatures and clinical factors as input in the training cohort. The optimal combinations of the radiomic signature and clinical factors were determined by using the Akaike information criterion (AIC) and the associations with the outcome of MVI status.

#### Step 5: Radiomic Model Evaluation

The radiomic signatures and models were further tested on the independent validation cohort. Receiver operating characteristic (ROC) curve analysis was used to evaluate discriminative performance, and the AUC was used to quantify the discriminative efficacy of all models that were established. Multiple ROC curves were compared by DeLong test. The 95% CI, sensitivity, specificity, and accuracy of each AUC was calculated.

Feature selection, classifier modeling, and statistical analysis were conducted by R software (3.5.2), The mRMR algorithm and RF classifier are described in Supplements B,C.

## Results

### Feature Selection and Classifier Modeling

From each ROI, a total of 1,595 radiomic features were extracted. Pair-wise Pearson correlation coefficients were calculated at both the classifier #1 stage and classifier #2 stage. The threshold for identifying highly correlated feature pairs was set at 0.9. As a result, 311 and 331 features from GTR and PTR remained at the classifier #1 stage. Subsequently, 282 GTR features and 107 PTR features were selected as input for the classifier #2 stage. The remaining features were ranked by mRMR. As a result, the top-100 features were selected for the classifier.

### Radiomic Model Establishment

By using the top-ranked features, the RF classifiers were trained on the training cohorts, which ranked from 2 to 100 with increments of 1 via mRMR to develop ultrasound radiomic signatures. The discriminative abilities of the ultrasound radiomic signatures were tested on independent validation cohorts, and the optimal signature with the best AUC was selected.

For the classifier #1 stage, the optimal signatures were obtained by combining the top-44 features selected for GTR (AUC = 0.708), and the top-25 features were selected for PTR (AUC = 0.710). The GPTR radiomics features extracted from the combined region of GRT and PTR showed AUC value of 0.680. The ultimate GPTR radiomic signature developed by logistic regression showed an increased AUC value of 0.726.

For the classifier #2 stage, the optimal signatures were obtained by combining the top-65 features selected for GTR (AUC = 0.806), and the top-80 features were selected for PTR (AUC = 0.752). The GPTR radiomics features extracted from the combined region of GRT and PTR showed AUC value of 0.742. The ultimate GPTR radiomic signature showed an AUC value of 0.770. The performances of all radiomic signatures are shown in Table 2. The formulas of GPTR signatures are shown in Supplement D.

**Table 2:** The performance of radiomic signatures.

**Classifier stage**	**Signature**	**AUC**	**95%CI**	**ACC**	**SEN**	**SPE**
Classifier #1	GTR	0.708	0.603, 0.812	0.624	0.784	0.531
	PTR	0.710	0.609, 0.811	0.653	0.757	0.594
	GPTR_(1)_	0.726	0.625, 0.827	0.663	0.838	0.562
	GPTR_(2)_	0.680	0.574, 0.786	0.634	0.811	0.531
Classifier #2	GTR	0.806	0.667, 0.944	0.730	0.333	0.800
	PTR	0.752	0.583, 0.921	0.757	0.333	0.929
	GPTR_(1)_	0.770	0.616, 0.923	0.730	0.667	0.750
	GPTR_(2)_	0.742	0.578, 0.906	0.649	0.778	0.607

*AUC, area under the curve; CI, confidece interval; ACC, accuracy; SEN, sensitivity; SPE, specificity; GTR, gross tumor region; PTR, peritumoral region; GPTR, Gross and peri tumoral volume*.

*GPTR_(1)_, the GPTR signature developed by logistic regression using GTR and PTR signatures*.

*GPTR_(2)_, The GPTR radiomic signature developed by radiomic features extracted from GTR and PTR combination region*.

### Radiomic Model Evaluation

**Figure 3:**
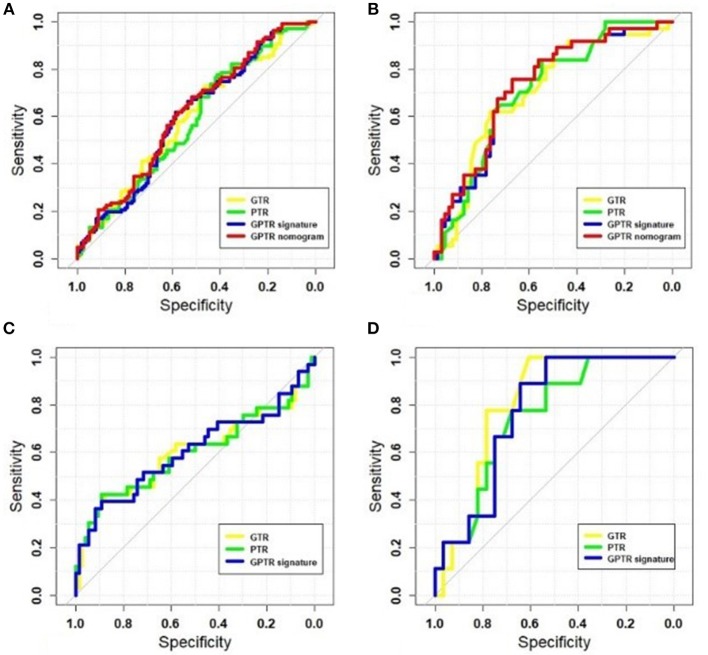
The receiver operating characteristic (ROC) curves of radiomic signatures and optimal nomograms. The following are shown: training cohort at the classifier #1 stage **(A)**; validation cohort at the classifier # 1 stage **(B)**; training cohort at the classifier #2 stage **(C)**; and validation cohort at the classifier #2 stage **(D)**.

The radiomic algorithm incorporating the optimal radiomic signatures and clinical factors showed better AUCs in comparison with those from radiomic signatures in the validation cohort. For the classifier #1 stage, after adding the AFP value, the AUC of the radiomic nomogram that combined the GPTR signature and the AFP value had an improved AUC of 0.744. The GTR and PTR radiomic nomograms that combined the radiomic signature and AFP were also evaluated, as shown in Table 3. The ROC curves in the training and validation cohorts—including those for GTR, PTR, and GPTR radiomic signatures of the GPTR algorithm— were shown in Figures 3A,B.

**Table 3:** Formulas and performances of the models.

**Classifier stage**	**Formulas**	**AUC**	**95%CI**	**ACC**	**SEN**	**SPE**
Classifier #1	0.327*GTR+0.375*AFP-0.043	0.723	0.622, 0.825	0.564	0.919	0.359
	0.271*PTR+0.368*AFP-0.044	0.739	0.642, 0.836	0.554	0.946	0.328
	0.334*GPTR+0.355*AFP-0.044	0.744	0.646, 0.841	0.634	0.892	0.484

*AUC, area under the curve; CI, confidence interval; ACC, accuracy; SEN, sensitivity; SPE, specificity; GTR, gross tumor region; PTR, peritumoral region; GPTR, Gross and peri tumoral volume*.

However, for classifier #2, none of the clinical factors were independently associated with MVI status. Figures 3C,D show the ROC curves for GTR, PTR, and GPTR radiomic signatures in both training and validation cohorts. The corresponding sensitivity, specificity and accuracy values for each classifier stages were calculated. The AUC of various radiomic models at classifier #1 and #2 stages were compared and the result of the DeLong test for the two-stage classifier is shown in Supplement E.

## Discussion

Successful preoperative assessment of MVI may facilitate patient management and improve survival (6, 9). Currently, assessment of MVI can only be achieved by histopathological examination after surgery. Subjectivity and sampling error are proved to be potential problems in accurately evaluating MVI (5). A non-invasive imaging method which could accurately diagnosing MVI preoperatively would be help to better stratify HCC patients for clinical management (38). Extensive studies have shown that radiomics have great potential in predicting tumor biology and in improving implementation of precision medicine (18, 23, 28, 29). Previously, some studies have established radiomic signatures for detecting the presence of MVI based on CT and MRI (11–14). Radiomic signatures based on arterial phase and delay phase of contrast-enhanced CT have yielded AUCs of 0.684 and 0.490, respectively (11). Additionally, radiomic signatures based on hepatobiliary-phase T1-weighted MRI have yielded an AUC of 0.705 in predicting MVI (14). A recent study incorporating clinical risk factors into ultrasound radiomic scores yielded efficacious performance in MVI prediction, with an AUC of 0.731 (18). Similarly, in our present study, based on a feature-ranking algorithm and classifier, we successfully established six grayscale ultrasound radiomic signatures to predict MVI status in HCC patients. The radiomic signatures based on features of GTR, PTR, and GPTR showed AUC values of 0.708, 0.710, and 0.726, respectively. When these radiomic signatures were combined with clinical factors in the radiomic algorithm, the performances of the GTR, PTR, and GPTR signatures at the classifier #1 stage were significantly improved, which demonstrated the added value of clinical factors in grayscale-ultrasound-based radiomic algorithms for individualized MVI prediction in HCC. On the training cohort, a model based on AFP values was further obtained by logistic regression. The model was tested on the validation cohort with an AUC value of 0.585. The GPTR signature showed an AUC of 0.726, which demonstrated that the classifier performance of the radiomic signature was better than that of a model built on AFP values. As a result, the nomogram built on both radiomic signatures and AFP values showed the highest AUC of 0.744. Hence, AFP and machine-learning-derived knowledge were mutually complementary. Comparing with CT or MRI imaging modalities, ultrasound is the most widely used first line imaging modality for diagnosis of focal liver lesions, with advantages as real time, no radiation exposure or nephrotoxicity. Meanwhile, the radiomics model based on ultrasound images also faces some challenges, such as limited resolution, relatively lower accuracy, highly operator dependent and flexible image scanning and record protocol.

Previously, various research on preoperative identifying MVI by imaging modalities has been mainly focused on inside tumor features. In recent years, imaging features of peri-tumoral area have been proved to be more accurate (18), since peri-tumoral tissue is the first area to be invaded by MVI (31). A high level of placental growth factor (PlGF) and expression of vascular endothelial growth factor receptor (VEGFR-1) in peri-tumoral tissue has been associated with peri-tumoral MVI pathological angiogenesis and potential vascular invasion (39). Therefore, imaging features involving peri-tumoral area may reveal a more direct association with MVI. A recent meta-analysis focused on the association between peri-tumoral MRI features and MVI, which revealed a significant association between MVI and peri-tumoral enhancement and peritumoral hypointensity on hepatobiliary-phase MRIs. However, the diagnostic accuracy analysis of this previous study showed relatively high specificity (0.90–0.94), low sensitivity (0.29–0.40) in assessing MVI (31). In another study, three radiomic models were built by extracting radiomic features from both intra-tumoral and peri-tumoral regions of Gd-EOB-DTPA-enhanced MRI images, which yielded an AUC value of 0.83 in predicting MVI (23). Until now, no study has ever extracted PTR radiomic signatures based on grayscale ultrasound for predicting MVI status. In our current study, we made a further comparison between intra-tumoral and peri-tumoral radiomic signatures. As our results showed at the classifier #1 stage, the grayscale-ultrasound-based radiomic features of GTR and PTR were both able to discriminate MVI status in HCC patients. The performance of the PTR signature was superior than that of the GTR signature. By combining the PTR and GTR radiomic signatures, the final GPTR radiomic signature performed better than GTR or PTR radiomic signatures in discriminating MVI-negative and MVI-positive cases. Additionally, at the classifier #2 stage, the GTR signature performed better than the PTR signature in further discriminating between M1 and M2 levels. By analysis of grayscale ultrasound radiomic signatures on peri-tumoral tissue in HCC patients, preoperative MVI assessment may become more accurate and reliable. Numerous methods could be used to develop GPTR signature. In our results, GPTR signatures obtained by logistic regression performed better than those obtained by radiomic features. Since different application scenarios will apply to different methods, in our future study, we will compare different methods in obtaining GPTR signatures based on larger image data.

Radiomic features based on imaging reflect the microscopic structure and biological behavior of the tumor, which has a direct relation to intra-tumoral heterogeneity (18, 40). Intra-tumoral heterogeneity may be associated with early microvascular invasion or a worse prognosis (41, 42). The trends of precision medicine in treatment of HCC are determined by genomic and biological characteristics of tumors, various imaging modalities represents a solution to elucidate these characteristics (4, 42, 43). It is difficult to clarify the correlation between a single radiomic feature with biological MVI behavior by selecting signatures from thousands of radiomic features. The common approach is to build a multi-feature parameter for radiomic analysis (44). Several studies have indicated that adding of mRMR can improve the performance of radiomic models (38, 45, 46). In our present study, the mRMR feature-ranking algorithms were added before the generation of radiomic signatures. The wavelet features showed strong abilities to predict other factors based on different modalities (47). Wavelet features were the primary method used in our study in optimizing GTR and PTR radiomic signatures at the two classifier stages (Supplement F), which can quantify potential heterogeneity at different scales of HCC lesions.

The present study has several limitations. First, the possibility of a selection bias cannot be excluded due to the retrospective nature of our present study. Secondly, our study was performed in a single center, although nine ultrasound machines were employed and distributed among the training and validation cohorts in our study, further multicenter validation might be necessary to evaluate the reliability and verify the generalization ability of our model. In addition, the number of patients with MVI-positive HCC lesions was relatively small. In the future, multimodality ultrasound imaging—including color Doppler-flow imaging, ultrasound elastography, and CEUS imaging—will be combined to improve the performance of MVI classification. We will also directly establish a three-classification radiomics model to distinguish the MVI-negative, M1, and M2 groups.

In conclusion, GTR and PTR radiomic signatures based on grayscale ultrasound imaging have potential value to facilitate preoperative prediction of MVI in HCC patients. Additionally, the GTR radiomic signature may be helpful for further discriminating between M1 and M2 levels among MVI-positive patients.

## Data Availability Statement

All datasets generated for this study are included in the article/Supplementary Material.

## Ethics Statement

The studies involving human participants were reviewed and approved by this retrospective study was approved by the institutional review board of Zhongshan Hospital, Fudan University. The patients/participants provided their written informed consent to participate in this study.

## Author Contributions

W-PW and XG: the respective roles of each author including study design and supervision. YD and QZ: ultrasound image acquirement and segmentation. LZ, WX, X-YZ, and J-MJ: radiomics analysis. All authors contributed to and agreed on the content of the manuscript. Each author participated sufficiently in the paper and approved the manuscript for submission.

### Conflict of Interest

The authors declare that the research was conducted in the absence of any commercial or financial relationships that could be construed as a potential conflict of interest.

## Abbreviations

HCC: Hepatocellular carcinoma
MVI: Microvascular invasion
GTR: Gross-tumoral region
PTR: Peri-tumoral region
GPTR: Gross- and peri-tumoral region
mRMR: Minimum redundancy maximum relevance
RF: Random forest.
