# Supplementary material for: Preoperative Prediction of Microvascular Invasion in Hepatocellular Carcinoma: Initial Application of a Radiomic Algorithm Based on Grayscale Ultrasound Images
Source: Front Oncol. 2020 Mar 19;10:353. doi: 10.3389/fonc.2020.00353 (PMC7096379; doi:10.3389/fonc.2020.00353)
Supplement: Supplementary file 1 [file Data_Sheet_1.pdf]

Supplement A: Radiomic features extraction

For each patient, the gross tumor region (GTR) was determined by delineating tumor contours based on gray scale ultrasound images using Medical Imaging Interaction Toolkit (MITK) software (version 2013.12.0; http://www.mitk.org/).

Radiomic features of 7 feature classes were extracted in 8 different image types. The shape features were only extracted in original image. So, the original image has 107 features, and each type image that underwent square, square root, logarithm, exponential and gradient filter has 93 features. We applied three dimensional wavelet transform to GTR and PTR. The three dimensional wavelet transform decomposed the original ROI into eight decompositions. Let L and H be the low-pass and the high-pass filtering, the wavelet decompositions of volume can be labeled as wavelet_LLL, wavelet _LLH, wavelet _LHL, wavelet _LHH, wavelet _HLL, wavelet _HLH, wavelet _HHL and wavelet _HHH. For instance, wavelet _HHL is obtained from x-directional high-pass filtering, y-directional high-pass filtering, and z-directional low-pass filtering of ROI. The obtained decompositions have the same size of the original image. For each of the eight decompositions, we computed the all the features except shape features, thus the 745 features were obtained from wavelet image. So far, 1595 radiomic features were obtained.

All features were listed as follows:

1) Shape features: Elongation, Flatness, Least Axis Length, Major Axis Length, Maximum 2D Diameter Column, Maximum 2D Diameter Row, Maximum 2D Diameter Slice, Maximum 3D Diameter, Mesh Volume, Minor Axis Length, Sphericity, Surface Area, Volume Ratio, Voxel Volume.
2) First-order statistical features: 10 Percentile, 90 Percentile, Energy, Entropy, Inter quartile Range, Kurtosis, Maximum, Mean Absolute Deviation (MAD), Mean, Median, Minimum, Range, Robust Mean Absolute Deviation (rMAD), Root Mean Squared (RMS), Skewness, Total Energy, Uniformity, Variance.
3) Gray Level Co-occurrence Matrix (GLCM) features: Auto correlation, Cluster Prominence, Cluster Shade, Cluster Tendency, Contrast, Correlation, Difference

Average, Difference Entropy, Difference Variance, Inverse Difference (ID), Inverse Difference Moment (IDM), Inverse Difference Moment Normalized (IDMN), Inverse Difference Normalized (IDN), Informational Measure of Correlation 1 (Imc1), Inverse Difference Normalized (IDN), Informational Measure of Correlation 2(Imc2), Inverse Variance, Joint Average, Joint Energy, Joint Entropy, MCC, Maximum Probability, Sum Average, Sum Entropy, Sum Squares.

4) Gray Level Run Length Matrix (GLRLM) features: Gray Level Non Uniformity (GLN), Gray Level Non Uniformity Normalized (GLNN), Gray Level Variance (GLV), High Gray Level Run Emphasis (HGLRE), Long Run Emphasis (LRE), Long Run High Gray Level Emphasis (LRHGLE), Long Run Low Gray Level Emphasis (LRLGLE), Low Gray Level Run Emphasis (LGLRE), Run Entropy, Run Length Non Uniformity (RLN), Run Length Non Uniformity Normalized (RLNN), Run Percentage, Run Variance, Short Run Emphasis (SRE), Short Run High Gray Level Emphasis (SRHGLE), Short Run Low Gray Level Emphasis (SRLGLE).
5) Gray-level size zone matrix (GLSZM) features: Gray Level Non Uniformity (GLN), Gray Level Non Uniformity Normalized (GLNN), Gray Level Variance (GLV), High Gray Level Zone Emphasis (HGLZE), Large Area Emphasis (LAE), Large Area High Gray Level Emphasis (LAHGLE), Large Area Low Gray Level Emphasis (LALGLE), Low Gray Level Zone Emphasis (LGLZE), Size Zone Non Uniformity (SZN), Size Zone Non Uniformity Normalized (SZNN), Small Area Emphasis (SAE), Small Area High Gray Level Emphasis (SAHGLE), Small Area Low Gray Level Emphasis (SALGLE), Zone Entropy, Zone Percentage, Zone Variance.
6) Gray Level Dependence Matrix (GLDM) features: Dependence Entropy, Dependence Non Uniformity (DN), Dependence Non Uniformity Normalized (DNN), Dependence Variance, Gray Level Non Uniformity (GLN), Gray Level Variance (GLV), High Gray Level Emphasis (HGLE), Large Dependence Emphasis (LDE), Large Dependence High Gray Level Emphasis (LDHGLE), Large Dependence Low Gray Level Emphasis (LDLGLE), Low Gray Level Emphasis (LGLE), Small Dependence Emphasis (SDE), Small Dependence High Gray Level Emphasis (SDHGL), Small Dependence Low Gray Level Emphasis (SDLGLE).

7) Neigbouring Gray Tone Difference Matrix (NGTDM) features: Busyness, Coarseness, Complexity, Contrast, Strength.

All radiomic features were normalized (z-score). The feature-extraction algorithm was developed and modified on an open access program PyRadiomics (https://github.com/Radiomics/pyradiomics) and performed within the setting of PyCharm Community Edition 2018.2.5

Supplement B: Minimum redundancy maximum relevance (mRMR)

Feature selection is an important procedure before constructing the classifier. Minimum redundancy maximum relevance (mRMR) is a particularly fast feature selection algorithm that attempts to select features have the maximal correlation with the class and simultaneously minimal redundancy to the already selected features (1). Both the relevance and redundancy are quantified by the mutual information (MI) defined as follows:

$$Y = I(x_i, h) - \frac{1}{|S|} \sum_{x_i \in S} I(x_i, x_j)$$

Where I is the MI, S is the set of selected features, and h is the class of label. The first term describes the relevance between the feature and class label, and the second term is the feature redundancy in the selected features set. Finally, the features which highly relevant to the class label and low redundancy with S were selected. In our study, the top 100 radiomic features based on mRMR were selected for GTR and PTR at two-stage classifier.

Supplement C: Random Forest Classifier

Random Forest (RF) is an ensemble of unpruned classification or regression trees created by using training data and random feature selection in tree induction (2-4). In this study, RF was implemented by the R package "randomForest" with tuning the parameter "ntree" varied from 100 to 500 with an increment of 10.

Supplement D: The formulas of GPTR signature in two-stage classifier（Table S1）.

Table S1. The formulas of GPTR signatures

Classifier stage	GPTR formulas
**Classifier #1**	1.931GTR signature+1.051PTR signature-1.517
**Classifier #2**	1.067GTR signature+2.734PTR signature-2.109

Supplement E: P value from DeLong test on training and validation cohort for two-stage classifier（Table S2 – S5）.

Table S2. *P* value from DeLong test on training cohort at classifier #1 stage

Model	GTR	PTR	GPTR	GPTR Nomogram
**GTR**		0.962	0.389	0.196
**PTR**	0.962		0.667	0.537
**GPTR**	0.389	0.667		0.236
**GPTR Nomogram**	0.196	0.537	0.236	

Table S3. *P* value from DeLong test on validation cohort at classifier #1 stage

Model	GTR	PTR	GPTR	GTR Nomogram
**GTR**	-	0.556	0.566	0.621
**PTR**	0.556	-	0.649	0.328
**GPTR**	0.566	0.649	-	0.294
**GTR Nomogram**	0.621	0.328	0.294	-

Table S4. *P* value from DeLong test on training cohort at classifier #2 stage

Model	GTR	PTR	GPTR
**GTR**	-	0.819	0.707
**PTR**	0.819	-	0.703
**GPTR**	0.707	0.703	-

Table S5. *P* value from DeLong test on validation cohort at classifier #2 stage

Model	GTR	PTR	GPTR
**GTR**	-	0.512	0.463
**PTR**	0.512	-	0.830
**GPTR**	0.463	0.830	-

Supplement E: Image features of the optimal radiomic signatures and formulas of GPTR signatures.

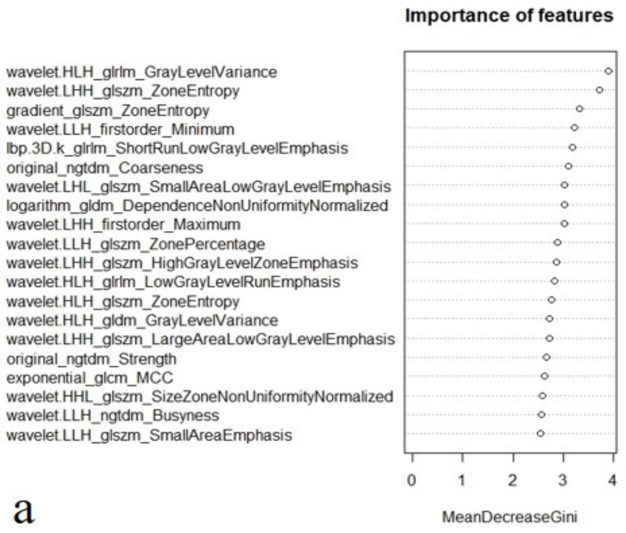

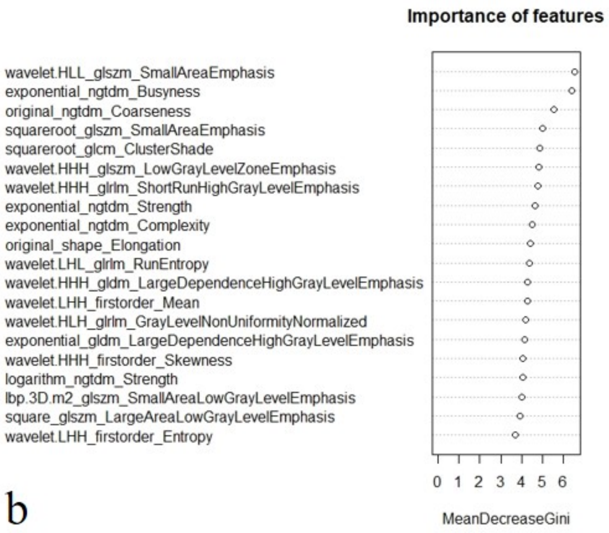
Importance of features
wavelet.HLL_glszm_SmallAreaEmphasis
exponential_ngtdm_Busyness
original_ngtdm_Coarseness
squareroot_glszm_SmallAreaEmphasis
squareroot_glcm_ClusterShade
wavelet.HHH_glszm_LowGrayLevelZoneEmphasis
wavelet.HHH_glrlm_ShortRunHighGrayLevelEmphasis
exponential_ngtdm_Strength
exponential_ngtdm_Complexity
original_shape_Elongation
wavelet.LHL_glrlm_RunEntropy
wavelet.HHH_gldm_LargeDependenceHighGrayLevelEmphasis
wavelet.LHH_firstorder_Mean
wavelet.HLH_glrlm_GrayLevelNonUniformityNormalized
exponential_gldm_LargeDependenceHighGrayLevelEmphasis
wavelet.HHH_firstorder_Skewness
logarithm_ngtdm_Strength
lbp.3D.m2_glszm_SmallAreaLowGrayLevelEmphasis
square_glszm_LargeAreaLowGrayLevelEmphasis
wavelet.LHH_firstorder_Entropy
0 1 2 3 4 5 6
MeanDecreaseGini

b

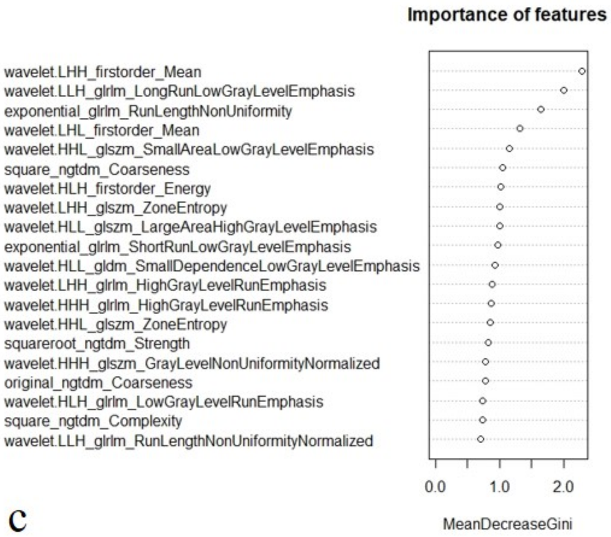
Importance of features
wavelet.LHH_firstorder_Mean
wavelet.LLH_glrlm_LongRunLowGrayLevelEmphasis
exponential_glrlm_RunLengthNonUniformity
wavelet.LHL_firstorder_Mean
wavelet.HHL_glszm_SmallAreaLowGrayLevelEmphasis
square_ngtdm_Coarseness
wavelet.HLH_firstorder_Energy
wavelet.LHH_glszm_ZoneEntropy
wavelet.HLL_glszm_LargeAreaHighGrayLevelEmphasis
exponential_glrlm_ShortRunLowGrayLevelEmphasis
wavelet.HLL_gldm_SmallDependenceLowGrayLevelEmphasis
wavelet.LHH_glrlm_HighGrayLevelRunEmphasis
wavelet.HHH_glrlm_HighGrayLevelRunEmphasis
wavelet.HHL_glszm_ZoneEntropy
squareroot_ngtdm_Strength
wavelet.HHH_glszm_GrayLevelNonUniformityNormalized
original_ngtdm_Coarseness
wavelet.HLH_glrlm_LowGrayLevelRunEmphasis
square_ngtdm_Complexity
wavelet.LLH_glrlm_RunLengthNonUniformityNormalized
0.0 1.0 2.0
MeanDecreaseGini

c

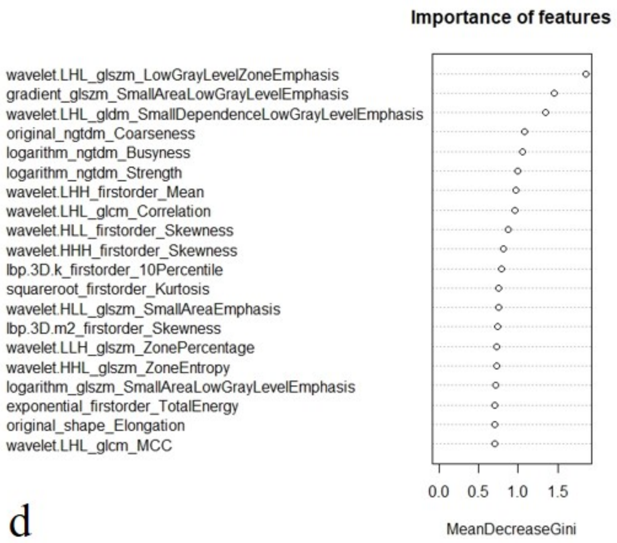
Importance of features
wavelet.LHL_glszm_LowGrayLevelZoneEmphasis
gradient_glszm_SmallAreaLowGrayLevelEmphasis
wavelet.LHL_gldm_SmallDependenceLowGrayLevelEmphasis
original_ngtdm_Coarseness
logarithm_ngtdm_Busyness
logarithm_ngtdm_Strength
wavelet.LHH_firstorder_Mean
wavelet.LHL_glcm_Correlation
wavelet.HLL_firstorder_Skewness
wavelet.HHH_firstorder_Skewness
lbp.3D.k_firstorder_10Percentile
squareroot_firstorder_Kurtosis
wavelet.HLL_glszm_SmallAreaEmphasis
lbp.3D.m2_firstorder_Skewness
wavelet.LLH_glszm_ZonePercentage
wavelet.HHL_glszm_ZoneEntropy
logarithm_glszm_SmallAreaLowGrayLevelEmphasis
exponential_firstorder_TotalEnergy
original_shape_Elongation
wavelet.LHL_glcm_MCC
0.0
0.5
1.0
1.5
MeanDecreaseGini
d

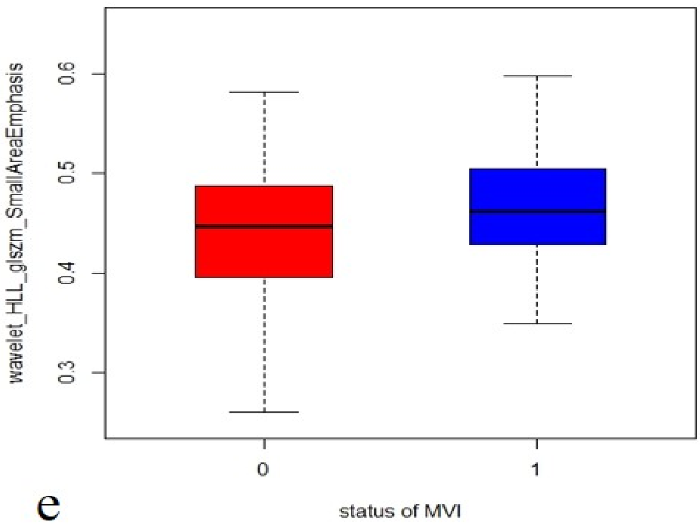
wavelet_HLL_glszm_SmallAreaEmphasis
0.3
0.4
0.5
0.6
0
1
status of MVI
e

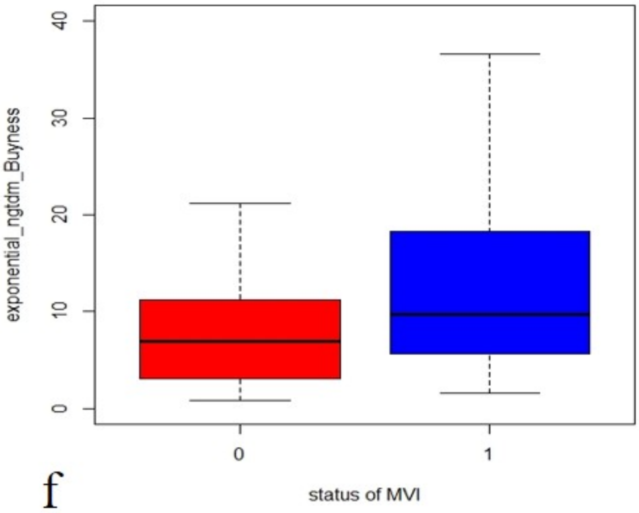

f

Figure S1. The top twenty features of each radiomic signature. (a) The GTR signature at classifier #1 stage, (b) The PTR signature at classifier #1 stage, (c) The GTR signature at classifier #2 stage, (d) The PTR signature at classifier #2 stage, (e) The boxplot of wavelet_HLL_glszm_SmallAreaEmphasis extracted from PTR, (f) The boxplot of exponential_ngtdm_Buyness extracted from PTR.

References

1. De Jay N, Papillon-Cavanagh S, Olsen C, El-Hachem N, Bontempi G, Haibe-Kains B. mRMRe: an R package for parallelized mRMR ensemble feature selection. Bioinformatics 2013;29:2365-2368.

2. Breiman L. Random forests. Machine learning 2001;45:5-32.

3. Liaw A, Wiener M. Classification and regression by randomForest. R news 2002;2:18-22.

4. Fernández-Delgado M, Cernadas E, Barro S, Amorim D. Do we need hundreds of classifiers to solve real world classification problems? The Journal of Machine Learning Research 2014;15:3133-3181.
